# Supplementary material for: The Southern Ocean diatom Pseudo-nitzschia subcurvata flourished better under simulated glacial than interglacial ocean conditions: Combined effects of CO2 and iron
Source: PLoS One. 2021 Dec 10;16(12):e0260649. doi: 10.1371/journal.pone.0260649 (PMC8664213; doi:10.1371/journal.pone.0260649)
Supplement: S1 File — Trace metal (TM) quotas without oxalate (Total TM content) and with oxalate wash (Intracellular TM content) determined at the end of the experiment in the four treatments of P. subcurvata (+Fe 190, Control 190, +Fe 290 and Control 290). The values represent the means ± SD (n = 3). Different letters indicate significant (p < 0.05) differences between treatments. (DOCX) [file pone.0260649.s001.docx]

***Pseudo-nitzschia subcurvata* flourished better under simulated glacial than interglacial ocean conditions**

Anna Pagnone^1^, Florian Koch^1^, Franziska Pausch^1^ and Scarlett Trimborn^1^

^1^ EcoTrace, Alfred Wegener Institute, Helmholtz Centre for Polar and Marine Research, Bremerhaven, Germany

## Cellular trace metal quotas

To determine intracellular TM quotas (Fe, Mn, Zn, Cu, Co) phytoplankton cells were collected on a 0. 2 µm TM clean PC filter at the end of the experiment. To remove the metal adhered to the cells’ surface, the filters were washed for at least 15 minutes with an oxalate wash and afterwards rinsed with natural Antarctic seawater (Hassler and Schoemann 2009). The rinsed filters were placed into TM clean 25 mL poly (fluor alkoxy) vials. The filters were digested in 5 mL HNO_3_ (distilled 65%, p.a., Merck) and 0.5 mL HF (40%, suprapure, Merck) after Ho et al. (2003) and Twinnig and Baines (2013). After the addition of 0.5 mL Milli-Q water, the cell extract was concentrated to 0.5 mL under a glass hood by evaporation at 140 °C. Subsequently, 0.2 mL HNO_3_ (distilled 65%, p.a., Merck) was added, the solution was moved to a 10 mL TM clean vial, 10 µL Rh (1 mg L^-1^) was added and the remaining volume of the vial was filled with Milli-Q water. The sample was than analyzed with an ICP-MS (Attom, Nu Instruments) to detect the intracellular Fe quotas, which were then normalized per filtered volume and per cell.

**Table S1:** **Trace metal** **(TM)** **quotas without oxalate (Total TM content) and with oxalate wash (Intracellular TM content) determined at the end of the experiment in the four treatments of *P.* *subcurvata* (*+Fe* *190*, *Control* *190*, *+Fe* *290* and *Control* *290*).** The values represent the means ± SD (n=3). Different letters indicate significant differences between treatments (p < 0.05).

| **Total TM content**  (amol cell^−1^) | ***P. subcurvata* incubations** | | | | |  |
| --- | --- | --- | --- | --- | --- | --- |
|  | **190** | | **290** | | |  |
|  | **+Fe** | **Control** | **+Fe** | | **Control** |  |
| Fe | 44.5 ± 12.7 ^a^ | 11.3 ± 1.8 ^b^ | 51.6 ± 9.1 ^a^ | | 22.1 ± 8.4 ^b^ |  |
| Co | 0.16 ± 0.01 ^a^ | 0.19 ± 0.07 ^a^ | 0.01 ± 0.01 ^b^ | | 0.1 ± 0 ^c^ |  |
| Cu | 3.78 ± 0.56 ^a^ | 3.03 ± 0.15 ^a^ | 3.06 ± 0.25 ^a^ | | 2.84 ± 0.40 ^a^ |  |
| Mn | 5.97 ± 0.64 ^a^ | 4.73 ± 0.39 ^a^ | 1.84 ± 0.35 ^b^ | | 3.14 ± 0.79 ^b^ |  |
| **Intracellular TM content**  (amol cell^−1^) | ***P. subcurvata* incubations** | | | | | |
|  | **190** | | | **290** | | |
|  | **+Fe** | **Control** | **+Fe** | | **Control** |  |
| Fe | 18.7 ± 8.7 ^a^ | 22.7 ± 4.7 ^b^ | 15.6 ± 9.0 ^a^ | | 7.4 ± 5.6 ^b^ |  |
| Co | 0.01 ± 0.04 ^a^ | 0.01 ± 0.01 ^a^ | 0 ± 0 ^a^ | | 0 ± 0 ^a^ |  |
| Cu | 0.34 ± 0.22 ^a^ | 0.51 ± 0.05 ^a^ | 0.22 ± 0.04 ^a^ | | 0.54 ± 0.21 ^a^ |  |
| Mn | 1.91 ± 0.14 ^a^ | 1.40 ± 0.13 ^b^ | 0.45 ± 0.10 ^c^ | | 0.52 ± 0.14 ^c^ |  |
