# Supplementary material for: The Southern Ocean diatom Pseudo-nitzschia subcurvata flourished better under simulated glacial than interglacial ocean conditions: Combined effects of CO2 and iron
Source: PLoS One. 2021 Dec 10;16(12):e0260649. doi: 10.1371/journal.pone.0260649 (PMC8664213; doi:10.1371/journal.pone.0260649)
Supplement: Attachment — Submitted filename: Response to reviewers_final.docx [file pone.0260649.s002.docx]

PONE-D-21-25243

The Southern Ocean diatom *Pseudo-nitzschia subcurvata* flourishes better under simulated glacial than interglacial ocean conditions

PLOS ONE

We thank the editor and the two reviewers for their kind evaluation and constructive criticism. Our response to each question are given in red and corresponding changes in the marked manuscript were highlighted in yellow and denoted with the line number (L#).

**Reviewer #1:**

In this well-written paper, the authors reported the ecophysiological responses of diatom *Pseudo-nitzschia subcurvata* to the simulated glacial and interglacial climatic conditions i.e., no Fe or Fe addition under 190 and 290 μatm pCO_2_ levels. They found the varied Fe and pCO_2_ levels had a limited effect on growth rate, but individually or interactively altered the cell compositions including pigments, POC, PON and BSi, as well as photophysiology of *P. subcurvata*. They conclude the combined higher Fe availability and lower pCO_2_ (present in glacial ocean) was beneficial for the diatom P. subcurvata, thus contributing more to primary production during glacial than interglacial times. There are some minor revisions should be considered before accepted for publication in PLoS One journal.

I think the title exaggerates the results of the experiment; because, the authors just simulated two environmental factors i.e., Fe and pCO_2_ levels in glacial and interglacial times, that cannot represent all the environmental factors.

We would like to keep the title as is, but have added to the opening paragraph of our discussion in L478-482: “*It is important to note that while we manipulated two of the main environmental parameters, Fe concentrations and CO_2_, other parameters (macronutrient concentrations, temperature i.e.) likely also differed between the glacial and interglacial ocean. For this study, however, the focus was on the interactive effects of Fe and pCO_2_”*.

In Discussion part, the authors fully explained the data in the view of physiology, some ecological perspectives should be given as they did the simulated field condition experiments.

We agree with the reviewer that it is always important to place the results of lab experiments in an ecological context. We have done this in the Conclusion (L641-653), which reads now: “*Assuming that P. subcurvata dominated phytoplankton blooms in the SO during glacial and interglacial times, we can conclude that P. subcurvata contributed more to primary production in the glacial than interglacial ocean. The higher POC production rates by the diatom under glacial conditions facilitated higher CO_2_ uptake from the atmosphere and potentially higher C export. This matches the ‘Iron Hypothesis’ of [1], which states that in the last glacial maximum higher Fe input from dust fertilized the SO, thus stimulating higher primary production and reducing thereby the atmospheric CO_2_ concentration. On the other hand, however, the thicker shells of P. subcurvata under the simulated interglacial conditions hint towards reduced grazing and thus its higher contribution to C export [107]. Biogeochemical cycles changed in the past and will change in response to future global climate change. Thus, understanding the dynamic interactions of the ocean’s biogeochemistry and phytoplankton is important in order to better simulate past and future climatic scenarios.”*

As the reviewer pointed out, we changed only the two parameters CO_2_ and Fe and thus we were careful not to exaggerate our results with grand statements.

1. Line 26, The ecological significance of “The thicker silica shells present under interglacial conditions” has been referred in the end of the Abstract; so, this sentence “which might offer better protection against grazers” should be removed. Besides, the authors did not give any descriptions about grazers throughout the manuscript.

As suggested by the reviewer, we removed this part of the sentence from the abstract in L26

2. Is there any geological evidence to show the Pseudo-nitzschia species dominate the Southern Oceans in glacial and inter-glacial times? I think some information should mentioned in the Introduction part.

This is a good point. We see no reason why *Pseudo-nitzschia* sp. would not have been present in the past ocean. Please note that literature on phytoplankton species composition in sediment cores is scarce. Often only diatom genera (i.e. *Fragilariopsis* sp., *Thalassiosira* sp. and *Chaetoceros* sp.) with thick frustules preserve well enough in SO sediment cores (i.e. Nair et al. 2015 Paleoceanogr & Paleoclim). As *Pseudo-nitzschia* *subcurvata* has a relative thin frustule, it is not well represented in the sediment record, but this does not automatically preclude that it was absent in the past ocean.

3. One of half brackets was missing in several places of the manuscript, e.g., Lines 105-106, Line 209, Line 220, Line 239;

We thank the reviewer for pointing out this formatting mistake. We have carefully gone through the manuscript to correct this.

4. In Materials and Methods part, clarify how much volume of culture was filtrated to measure the POC, PON, BSi and pigments.

For clarification, we added in L244-245 and in L256-257, respectively: “*The amount filtered ranged between 100-300 mL and was dependent on the biomass in the treatments*.” and “*The amount of seawater filtered to collect pigment samples ranged between 200-300 mL on the GF/F filter and was dependent on the biomass in the treatments.*”

5. In Line 195-196, the authors clarified the cell densities at the end of the cultivation were 67000 to 107000 cells per ml; I’m wondering what the cell density was at the initial inoculation. Please clarify it.

The aim of the initial inoculate was ~1000 cells mL^-1^. We added in L194-196: “*The main experiment started with initial cell densities of ~1000 cells mL^-1^, lasted between 8 and 9 days and reached final cell densities between 67 000 and 107 000 cells mL^-1^.*

6. Line 280, The light intensity of fluorometer is so high. Please check it.

We thank the reviewer for pointing this out since there was an error with the units. The umol was removed in L283. Other than that, this is the correct intensity. 1.2·10^22^ photons m^-2^ s^-1^, which is in line with the light setting recommended in the manual of the FastPro8 GUI and FRRf3 systems documentation by Kevin Oxborough (2012). But we thank the reviewer for pointing this out.

7. Line 252-253, Production rates of POC, PON and BSi were calculated by multiplying the cellular quotas with the respective growth rate. It’s OK to calculate PON and BSi production rate. But I don’t think it’s right to use this way to calculate POC production rate; because, the cultures were grown under 16:8 light:dark cycle under 100 µmol photons m-2 s-1 light intensity, that means the POC production by photosynthesis just occurs in light duration (16 h per day), while the growth rate was calculated from the cell density changes of a whole day (24 h). So, they are mismatching.

The reviewer is correct that the time of sampling and the interval used for normalizing rates plays a role, especially for photosynthesis and carbon fixation. Please note that production rates of POC, PON and BSi were normalized to the total incubation time of the respective treatment. This means, we compared the amount of POC, BSi and PON, which accumulated over the whole course of the experiment (between 8 and 9 days depending on the treatment). Hence, normalization of these processes over an entire diel cycle thus integrates the responses over both the light and dark phase and resultes in NET rates per day. This approach is also commonly used by other groups (i.e. Xu et al. 2014 Limnol Oceangr, Müller et al. 2015 MEPS, Peng et al. 2017 MEPS).

8. Line 337-338, The authors clarified that “dFe values denote two measurements”; so, it is wrong to use the standard deviation here. I suggest to replace the SD to half of range.

We have amended the table to show the two values obtained for each parameter for the culture media.

**Reviewer #2:**

The following manuscript provides details on the interactive effects of Fe and pCO2 on an Antarctic diatom, Pseudo-nitzschia subcurvata, under glacial and inter-glacial conditions. Whilst there is a wealth of evidence of understanding higher pCO2 concentrations, representative of future climate change, very little is known about how this diatom copes under these pre-industrial conditions. The manuscript is very well written, with clean concise results and figures. I recommend this paper for publication, with only minor corrections which are outlined below.

Minor Corrections

Line 248: Should the NaOH concentration not be “M”, currently it is “N”.

The reviewer is correct. We changed this in L249.

Line 276: Were the samples collected during the day or night part of the light cycle? This will greatly impact the effects of dark adaptation (see Schuback et al. 2021 Frontiers for more details on this).

We thank the reviewer for pointing out this publication. The samples were always collected at least 2 hours after begin of the light period. This is now mentioned in L278. We chose the length of the dark acclimation for NPQ-relaxation to be 10 minutes, a time based on our previous experiments with various cultures, including *P. subcurvata* (Trimborn et al. 2013, 2014, 2019; Koch et al. 2019).

Line 284: No text is provided to determine whether blanks were collected, and the fluorescence subsequently corrected. This is an important step for any active chlorophyll fluorescence measurements.

We added this in L280-281: “*For each treatment, a 0.2 μm filtered fluorescence blank was collected, measured and subtracted.”*

Line 294: Please provide the number of measurements per light level. Was any QC applied to these measurements per light level? As stated in reference 76 (Ralph and Gademann) unlike traditional PE curves, the measurements do not reach steady state. That is why previous studies have opted for either taking the mean of the last 3 measurements per light level or applying a QC to remove potential outliers.

The reviewer’s point is a good one and we have accounted for it in the text by adding this information in L297-300: “*At each light level, six measurements of the light-adapted minimum (F′) and maximum (F_m_′) Chl a fluorescence were taken and the effective PSII quantum yield (F_q_′/ F_m_′ = (F_m_′ - F′)/ F_m_′) was calculated.”*

Line 302: More details are required here to indicate how the curve fitting was done. Was this done in FastPro8 or was another program used to fit the curves.

As pointed out, the curve fitting was done according to Ralph & Gademann (2005). To clarify which software was applied for the curve fitting, it now reads in L317-319: “*All statistical analyses and the curve fittings were performed using the program SigmaPlot (Version 13.0 from Systat Software, Inc., San Jose California USA, www.systatsoftware.com).”*

Line 306: Reference required for the Stern-Volner equation – Bilger and Bjorkman, 1991, doi 10.1007/BF00197951.

As suggested, we now cite this reference in L310 and have added Bilger and Bjorkman (1991) to our reference list.

Line 329: This statement is a little confusing, based upon the significant differences above. Are you saying that the CO2 parameters are significantly different between 190 and 290? Or are you saying they are significantly different between the Fe and control? If the latter, what impact do you think it will have on the results if the CO2 parameters are also significantly different alongside the Fe concentrations.

We agree with the reviewer that the statement did not concisely represent what we wanted to bring across to the reader. We changed it in L331-336: “*As expected, the pCO_2_ and DIC in all of the 290 treatments were significantly higher than in the 190 treatments (2-way ANOVA; p<0.001; Table 1). Biologically driven changes to the carbonate chemistry were ruled out since TA, DIC, and pCO_2_ values did not differ for each treatment between the abiotic culture medium and the corresponding P. subcurvata incubations for each treatment at the end of the experiment (Table 1).”*

Line 339: Unless I have missed it, I am struggling to find where the definition of the letters is stated. This makes it difficult to understand what was significant or not.
From my understanding:

a = all the same

b = significantly different to a

c = significantly different to a and b

Perhaps you can make it a little clearer for the reader.

The reviewer is right, this is how the different letters need to be interpreted. To make this clearer, we amended the text in the legend and it now reads (L343-345): *Differences between the individual treatments of the P. subcurvata incubations were determined with post hoc tests, where significant statistical (p < 0.05) differences are denoted by different letters.”* We hope that this clarifies things for the readers.

Table 1: dFe concentrations – do you know what may have caused such differences? Additionally, a 1 nM concentration for the 190 control would not necessarily be limiting for SO phytoplankton species based upon measured in situ concentrations.

The reviewer is absolutely correct in that 1 nM is on the higher end of concentrations, which phytoplankton usually experiences in HNLC regions of the world’s ocean. However, the Fv/Fm in the 190 control was 21% lower than in the +Fe, which is a physiological response indicative of an iron-stressed *P. subcurvata* culture. This indicates that the slightly higher dFe concentrations in the 190 Control vs the 290 Control, likely happened during dFe sampling. Working with Fe is notoriously tricky and prone to contamination and thus we cannot rule out that this may be the reason for the higher dFe concentrations in the 190 Control vs the 290 Control.

Line 378: Do you mean “but not in 190”?

Thank you for pointing this out, accordingly we changed 290 to *190 in L383*.

Line 416: The connectivity parameter is most often reported as the Greek letter rho, ρ.
We thank the reviewer for pointing this out. We have always used P in our past publications. For consistency, we would like to stick with this nomenclature, especially in light of the fact that we use rho in our nutrient uptake kinetics work.

Table 4: Units of α, should this not be amol e- cell-1 s-1 (μmol photons m-2 s-1)-1?

We agree with the reviewer and have changed this in table 4.

Line 458: This does not make sense to me, in the 190 treatment the standard deviations are 9 and 11, whereas in the 290 treatments they are 15 and 9. So how is it that the large standard deviations in the 190 prevent statistical significance?

We agree with the reviewer that this was confusing and have changed the sentence to read (L462-463): “*Although not significant (p>0.05), in the 190 treatments a similar trend was observed.”*

Line 505: I wouldn't necessarily agree with this statement. The Chl:C ratios are similar for both Fe treatments at 190 and 290. As well as σPSII being similar between Fe and control at 290. These are better indicators of potential light absorption for photosynthesis.

We thank the reviewer for pointing this out and have rephrased this statement in L511-514: *“Such a negative CO_2_ effect in Fe-enriched conditions was also observed in the Chl a content (Fig. 2A) and the number of functional RCII (Table 4). Indeed, P. subcurvata cells grown in the +Fe 290 treatment had a lower Chl a content compared to ones in the +Fe 190 treatment (Fig. 2A), although the Chl a:C ratios were similar.”*

Line 540: Missing some more recent work: Strzepek et al. 2019 doi: 10.1073/pnas.1810886116

We have added this paper to the citations in L549 as well as in the reference list.

Line 553: See comment above - I do not think you can solely use Chl-a concentrations to infer reduced light absorption.

We agree with the reviewer that Chl a alone is not enough and have modified the sentence (L561-563): “*Because Fe deficiency affects the synthesis and thus cellular content of Chl a, as seen in our data (Fig. 2A), light harvesting may become more difficult for the cell.”*

Line 559: Your statements above do not agree with this. Here you state clearly mechanisms that help to maintain light absorption - but above you state there is less light being absorbed due to less Chl-a.

We agree and have changed the statement regarding Chl being solely responsible (see above, (L561-563).

Line 564: This statement is false. Ik is the inflection point of light-limiting versus light-saturating. Under Fe-deficiency Ik was higher, indicating that it took more light for the cells to become saturated.

The reviewer is correct that this statement was not accurate. We amended the sentence to read (L569-577): “*These photophysiological adjustments, however, did not prevent changes in light absorption completely of Fe-limited P. subcurvata (Table 4). Higher α values were found under Fe deficiency for both 190 and 290 treatments, indicating that Fe-deplete cells were more efficient of light capture at lower irradiances than Fe-replete cells. In addition to this, I_k_ remained either unchanged at 190 or was slightly higher at 290 (Table 4). In other studies, Ik either decreased [40,91] or remained unchanged [44,84] under Fe reduction. Thus, while Fe deficiency at 290 resulted in a more efficient light utilization at lower irradiances (higher α), the cells required more light (higher I_k_) in order to cover their photosynthetic requirement [40*].“

Line 579: I would recommend also calculating the normalised stern-volner NPQ as well and determining whether any differences can be seen here. NPQ stern-volner has been show to have poor correlation with other photophysiological metrics, whereas the normalised stern-volner examines changes in both the dark and light-regulated states. This makes it useful to comparing samples under different Fe conditions. See Schuback et al. 2021 Frontiers for more details.

We thank the reviewer for pointing out this new paper, which just was published, when our manuscript was submitted to PLOS One. Until now, we have always used NPQ according to equation 6 in L312 in this and previously published papers from our working group. Even though we agree that normalised stern-volmer NPQ values would be a nice addition to our manuscript, we, belief that this information would not help to clearly identify whether it was the PTOX pathway, which potentially acted as alternative electron sink. Therefore, we would like to keep the current part of the discussion as it is and will use this new equation when analyzing future photophysiological data sets, in particular when investigating Fe limitation effects.
